# Bridging the Gap: Enhancing HPV (Human Papillomavirus) Education to Combat Rising Cancer Rates

**DOI:** 10.7759/cureus.74023

**Published:** 2024-11-19

**Authors:** Riya A Sood, Beatrice G Carpo, Joerg R Leheste

**Affiliations:** 1 Biomedical Sciences, New York Institute of Technology College of Osteopathic Medicine (NYIT-COM), Old Westbury, USA

**Keywords:** adolescents, cancer, community-based approach, health policy, hpv, hpv-education, hpv-linked cancers, human papillomavirus, public health, u.s. congress

## Abstract

Human papillomavirus (HPV) infections are sexually transmitted and contribute significantly to the spread of cancers in both men and women, including oropharyngeal and cervical cancers. The shortcomings of the current preventative strategies are becoming increasingly obvious, underscoring the need for new approaches, especially in the area of health education. Information accessibility, cultural appropriateness, proper management of information accuracy, and the spread of misinformation are emerging as critical focal points for improving the situation. This health policy research review evaluates contributing factors, such as HPV policies, health literacy levels, cultural barriers, healthcare provider-to-patient education, school health education programs, and current awareness campaigns. Based on these findings, the study highlights the most effective strategy for providing actionable information to improve public health: a community-based, multifaceted, but integrated approach to HPV education. To achieve this goal, healthcare provider recommendations are absolutely critical in influencing vaccine decisions. Furthermore, physical and online information materials must be designed at the appropriate reading level, with cultural considerations in mind, and screened to aid comprehension and prevent the spread of misconceptions, stigmas, and misinformation.

## Introduction and background

**Figure 1:**
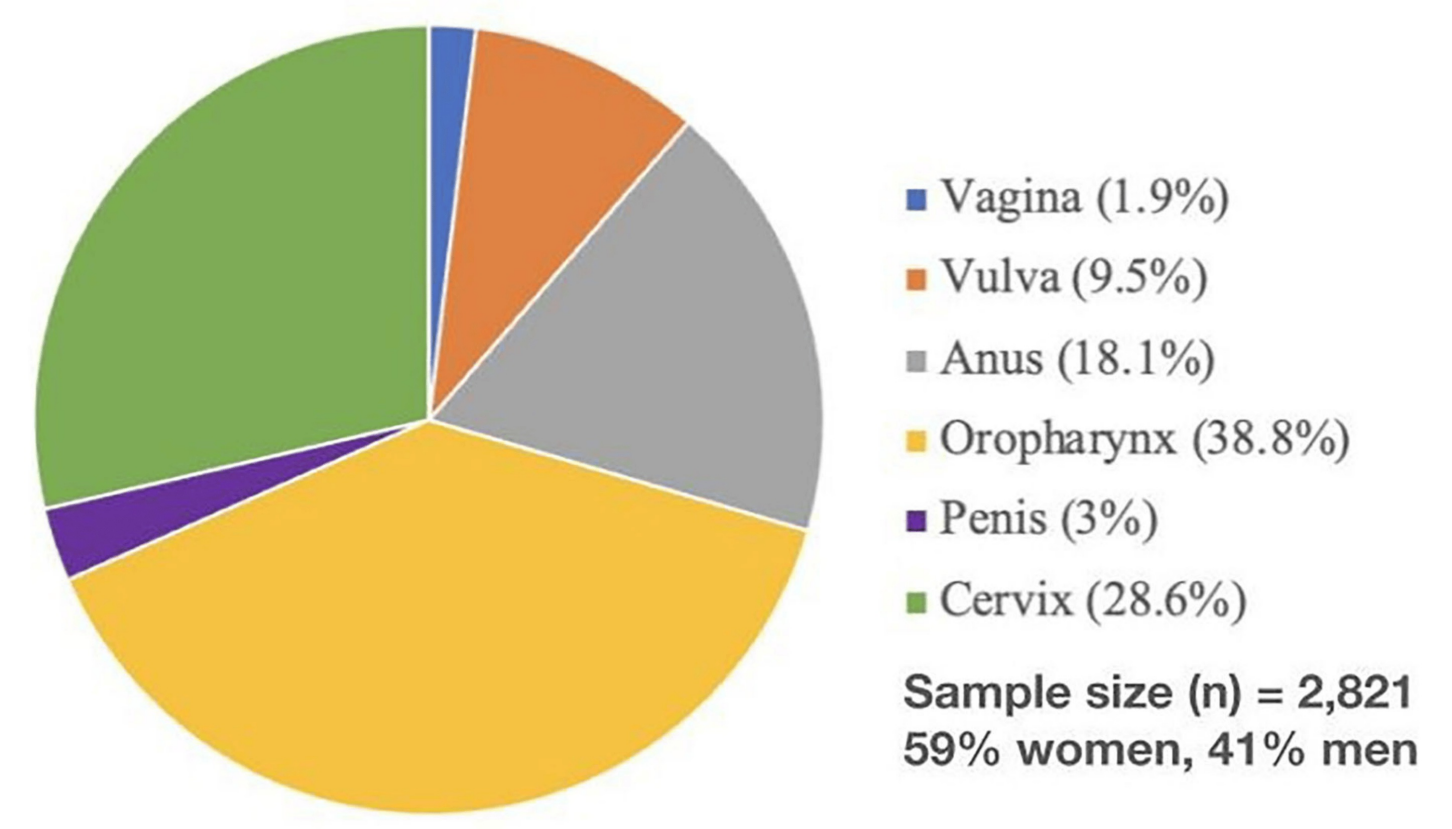
Proportions of the various types of HPV-related cancers between 2015 and 2019, according to the New York State Department of Health. Both men and women are affected by HPV-related cancers – women at a slightly higher rate. Men and women can develop oropharyngeal cancer and anus cancer, while other types are gender specific [2].

Human papillomavirus (HPV) is the most prevalent sexually transmitted virus and has the potential to cause cancers of the penis, anus, cervical region, vulva, vagina, and oropharynx [1,2] (Fig. 1).

HPV incorporates more than 200 different strains that fall into high- and low-risk types. High-risk strains, such as HPV16 and 18, cause cancer primarily through the production of two proteins, E6 and E7, that act as oncoproteins by suppressing the function of essential tumor suppressor proteins such as p53 and Rb, respectively. This can result in uncontrolled cell proliferation and cancer. When given as the Centers for Disease Control and Prevention (CDC) advises, the HPV vaccine has shown to be effective in preventing HPV and HPV-related diseases. Surprisingly, despite the availability of a wide range of interventions targeting HPV, an annual documentation of 37,300 new cases remains [3]. Furthermore, HPV is predominantly associated with cervical cancer, a disease that causes an estimated 4,000 deaths annually with an incidence of approximately 11,500 [1]. This raises the question of the source of the discrepancy between the actual state of HPV-transmitted diseases and the best potential medical outcome. Our research reveals that not enough people in the United States know about HPV and the cancers it can cause. The problem is multifaceted, but academic programs often don't have enough public service announcements (PSAs), written materials, and HPV discussions. Furthermore, the World Health Organization (WHO) reports that consultation services provided by healthcare providers and non-profit organizations are difficult to access [4]. In accordance with the WHO's objective of eradicating cervical cancers worldwide by the turn of the next century, it is vital to evaluate the most efficacious approaches to HPV health education. Moving forward, we must tailor educational resources to meet the needs of rural and at-risk communities. The information that is made public must be medically correct and cover all types of cancers linked to HPV, not just gynecologic cancer but also penis, anus, and oropharynx cancers [1]. By assessing current federal health policies, health literacy levels, and educational forums, we can create a better platform for disseminating objective, validated information that encourages male and female vaccination and limits HPV transmission.

Objectives

This health policy research brief is based on a narrative review of the literature with the goal of identifying and exposing potential educational gaps in HPV education. It investigates how disjointed, incomplete, and misleading HPV information affects HPV transmission and the incidence of associated diseases. This initiative aims to raise awareness about the importance of accurate HPV education, emphasizing that improved education can lead to enhanced public health outcomes. By reducing educational gaps, we can enhance informed decision-making and stakeholder engagement. That in turn will increase vaccination rates, reduce HPV transmission, and result in fewer cases of HPV-related malignancies, such as cancer. This could significantly reduce healthcare expenditures associated with treating chronic diseases while improving population health. An important aspect of this work is the identification and recommendation of actionable next steps for the United States Congress to consider, aimed at narrowing and resolving observed gaps in HPV education.

Methods

This narrative review was conducted over a nine-month period between December 2023 and September 2024. We employed a comprehensive search strategy to identify relevant literature on HPV awareness, education, misinformation, vaccination rates, and infection. Our search strategy focused on peer-reviewed articles on PubMed using a combination of Boolean operators. The complete list of search terms included: “human papillomavirus”, “HPV”, “awareness”, “education”, “misinformation”, “vaccination”, “infection”, and “rates”. The search was limited to studies published between 2010 and 2024 to ensure relevance to current practices and understanding. This search yielded 85 relevant studies, resulting in a total of 186 papers. Inclusion and exclusion criteria were applied by two independent reviewers to evaluate the identified studies based on their relevance to the following research questions: "Does misinformation about human papillomavirus lead to increased rates?", "Where does the educational gap lie?" Studies were included if they: addressed the above questions, were peer-reviewed articles, and involved human subjects. A total of 33 articles met these criteria and 52 studies were excluded for not directly addressing the research questions or for being outside the specified publication years. To facilitate categorization and data extraction, the 33 included articles were categorized into six themes: "politics and current policy", "health literacy", "cultural barriers", "healthcare provider education", "awareness campaigns", and "school education". For each study, we systematically extracted and recorded the following information in a shared spreadsheet for collaborative evaluation and validation by the principal investigator (PI): title, authors, study design, year of publication, interventions, results, and conclusions. To enhance transparency, we assessed the quality and risk of bias of the included studies. This assessment focused on study design, sample size, and methodological rigor, ensuring that our results and recommendations are primarily drawn from high-quality studies.

## Review

Background on HPV legislation

In 2006, the U.S. Food and Drug Administration (FDA) granted regulatory approval for the HPV vaccine [5]. In the five years that followed, 32 of the 141 state-level vaccine bills became law. These bills addressed legislation on various aspects of public funding, private insurance funding regulations, and the availability of vaccine information to parents and schools [5]. Despite significant efforts to promote HPV prevention and reduce HPV-induced cancer incidence, only 60% of adolescents have completed the HPV vaccination series [3]. Furthermore, estimates project that 37,300 new cases of HPV-related cancers will emerge each year under the current circumstances.

One interview-based study revealed that most Latino immigrant parents adhere to their pediatrician's advice during their children's annual well-child examinations, aged 9-10 [6]. However, many of these parents reported that their pediatrician did not even mention the HPV vaccine recommendation during their visits, suggesting the need for new policies. Meanwhile, a community-based approach to HPV education has demonstrated its effectiveness in increasing vaccination rates among cultural and religious communities [7]. These strategies should emphasize the importance of HPV vaccinations for both young boys and young girls, addressing and dissipating concerns that the vaccine may link to risky sexual behavior. To promote community health and disseminate educational materials about HPV and the vaccine's potential benefits, local communities, and political leaders must play an active role. However, the majority of experts concur that a physician's recommendation plays the most crucial role in determining vaccination decisions [3].

Impact of information quality and health literacy

When examining the relationship between information quality, delivery, and elevated HPV rates, it is critical to consider factors that may contribute to the incorrect interpretation of health data. Health literacy enables individuals to acquire, understand, and use health information to make informed decisions about HPV. Despite having a very basic understanding of HPV, a significant number of college students polled for a study believed they were immune to HPV infection, which may reflect the fact that HPV infection is frequently clinically asymptomatic [8]. According to this and related studies, most students are unaware that there is a significant chance they will acquire HPV within their lifetime. Every year, approximately 13 million Americans receive a new diagnosis of HPV infection [1]. This divergence between the theoretical potential of the virus and the tangible consequences it can impose on an individual is a major driver behind the current situation. Moreover, research shows that most college students are unaware that males can access HPV tests and that, apart from cervical cancer, HPV can also cause anal cancer, penile cancer, and head and neck cancer [8]. To address this knowledge gap, public health campaigns aimed at eradicating cervical cancers should include measures that specifically target male adolescents in addition to females [9].

Adolescents frequently express resistance to receiving the HPV vaccine for a variety of reasons, including not having their doctors recommend it, not knowing enough about it to make an informed decision about their health and wanting to be self-sufficient when it comes to matters such as vaccination [10,11].

It is common for medical professionals to use specialized language when recommending the HPV vaccine over other vaccinations due to the virus's complexity in terms of carcinogenic potential, as well as the importance of timing before the onset of sexual activity. Research has shown that patients often postpone receiving the HPV vaccine until a later appointment, limiting its effectiveness [12]. If we give this vaccine recommendation the same priority and urgency as other vaccinations, including a proper risk-benefit analysis, adolescents will gain a better understanding of the vaccine's doses and schedule. As a result, adolescents would be better able to make informed decisions about how to handle the situation.

The availability of information on the internet is arguably one of the most influential factors in modern society's decision-making, including medical decisions. Vaccines are no exception, as parents have the right to detailed information about the substances they administer to their children. Online information should present itself in simple, understandable language to assist parents in making informed decisions about the HPV vaccine. On the contrary, a simple internet search for HPV vaccination yields the majority of results at a reading proficiency level inappropriate for tenth graders, preventing many from reaching an informed decision [13,14].

Researchers have discovered a link between transgender and gender-diverse people searching for HPV and social media vaccine information, as well as a drop in vaccination rates. This is despite the fact that this group of people has higher overall vaccination rates than the general population [15]. Anti-vaccine organizations may specifically target transgender and gender diverse people, despite complex reasons for this observation. These groups frequently use existing vulnerabilities and mistrust of the healthcare system to spread their messages [16]. Furthermore, the rights and healthcare needs of transgender and gender-diverse people are frequently questioned on public and social media platforms, leading to widespread distrust of medical information disseminated through these channels [16].

Extent and impact of cultural hurdles

Cultural differences and belief systems that influence information assimilation and comprehension must be carefully considered when designing effective educational intervention tools. A variety of factors, including socioeconomic status, religious affiliations, and language barriers, can influence an individual's perspective on significant issues, potentially leading to stigmatization of the vaccine. For example, certain religious customs value chastity and prohibit sexual activity prior to marriage; this may lead to negative perceptions of the HPV vaccine, which is linked to sexual activity [17,18]. These misconceptions undermine the benefits of vaccination, contributing to lower-than-average vaccination rates, particularly among certain populations. Strong correlations between the HPV vaccine and cervical cancer, on the other hand, highlight the vaccine's importance for young girls in particular, whereas young boys do not show similar associations. As a result, men are less likely to start the HPV vaccine series [19]. Male vaccination, on the other hand, is critical not only because they are carriers of the virus and can spread it to others, but also because males are more susceptible to a variety of other HPV-related cancers.

Immigrants to the United States have consistently shown lower rates of initiating HPV vaccination series [19-21]. It is noteworthy that these populations frequently lack the HPV vaccine series, despite having strong predictors of engaging in preventative health behaviors elsewhere. This raises the question of how this gap came to exist. According to this study, language barriers to comprehension and understanding are the underlying cause. Thus, we should adapt communication to patient and parent comprehension. Higher HPV vaccination rates are expected to be facilitated by the ability to effectively communicate vaccination objectives in the primary language of patients and their parents, or in terminology that is easily understandable to them [17,21-22]. Furthermore, research has shown that individuals without health insurance or a primary care provider are among those who start the HPV vaccine series at a lower rate [19]. As a result, low-income communities have disproportionately high rates of HPV infection. Thus, it is critical that educational resources be readily available and developed in a comprehensible manner that emphasizes the risks associated with HPV-caused cancers [23].

Importance of healthcare provider training

According to studies, a persistent challenge in the effort to enhance vaccination rates is the lack of guidance or recommendations issued by healthcare professionals [24]. Healthcare settings don't have enough education and vaccination programs, and spreading false or incomplete information about HPV makes people in high-risk groups less likely to take advantage of chances to improve their health [25]. Individuals with limited access to healthcare and inadequate education had significantly lower levels of knowledge and attitudes about human papillomavirus (HPV) than the national average. Researchers discovered a correlation between low rates of current Pap test use and a lack of understanding of the link between HPV and abnormal Pap tests [26].

Additionally, healthcare providers' overestimation of parental reluctance to receive the vaccine, combined with insufficient parental knowledge about the vaccine, impedes vaccine acceptance. Many pediatricians still support standard childhood vaccines, but studies have shown that the HPV vaccine does not get as much support for 11-12-year-olds who are eligible. This is true even though healthcare providers know the CDC-recommended schedule for HPV vaccines between the ages of 11 and 12 (Table 1) [27].

**Table 1:** CDC HPV vaccination schedule Due to the varying benefits of the HPV vaccine in adults, individuals aged 27 to 45 should consider shared clinical decision-making (SCDM). Table modified from original source [27].

Indicated Age	Vaccination Strategy	Additional Indications/Precautions
11-12 years of age	Routine vaccination	Can start as early as 9 years of age
13-26 years of age	Catch-up vaccination	If not adequately vaccinated
27-45 years of age	Shared clinical decision-making	If not adequately vaccinated; depending on individual's health and other confounding factors

Pediatricians and family physicians unquestionably play an important role in disseminating information and education about HPV vaccination to the general public [28]. Nonetheless, gynecologists, dentists, otolaryngologists, and urologists have an additional responsibility to educate their patients about the critical importance of HPV prevention in the context of cancers related to their specialty.

Collaboration and interprofessional education, for example, are critical in combating the rising incidence of HPV-associated oropharyngeal cancers. Since many healthcare professionals don't like to talk about their patients' immunization status, it is important to do things to teach people more about the human papillomavirus (HPV) and encourage them to follow official organizational recommendations [29]. The inclusion of health coaches and patient navigators would be beneficial in improving health literacy and connecting patients and providers with HPV and cervical cancer services [26,30].

Providing healthcare providers with a systematic, standardized approach to patient education would improve their ability to educate patients about HPV. Studies have shown that interventions in healthcare facilities, like a cancer prevention awareness program, enhance the acceptance of the adolescent HPV vaccine. As a result, physicians could make it easier to communicate information and advice about HPV-related cancers by organizing existing HPV data into a useful framework [31]. Furthermore, reminder systems that link the HPV vaccine with other vaccinations, as well as strong endorsement of the vaccine as a safe preventive measure, can all contribute to increased vaccination rates and vaccine uptake [24-32].

A recent set of studies raised an interesting new perspective on the potential contribution of biases and judgment errors due to heuristics simplifying decision-making under conditions of uncertainty. An analysis of interviews with pediatric clinicians revealed biases such as anchoring, present, and optimism bias, which contribute to weaker vaccine recommendations, suggesting that increasing awareness of these biases could enhance clinician communication about HPV vaccination [33,34].

Role of schools in HPV vaccination

Despite attempts to increase HPV vaccination rates among school-aged children, determining the most effective strategy has proven difficult. According to the available literature, a number of factors, to varying degrees, impede the start and successful completion of the vaccine series, thereby impeding the entire vaccination process. Evidence suggests that implementing educational programs, fostering collaborative decision-making processes, and streamlining school operations did not significantly boost vaccination rates. However, requiring HPV vaccination for school entry would increase initiation rates and, most likely, follow-through [35]. This is an important point to emphasize, as schools serve as ideal venues for vaccine delivery due to their ability to reach a large number of individuals of various ages, particularly during years when attendance is mandatory. As a result, this strategy would encourage the elimination of vaccination access disparities and make it easier to achieve high vaccination coverage rates [36].

Nonetheless, even within educational settings, there is room for improvement. Even though there were a lot of people who started getting the vaccine, school-based health centers had a challenging time giving all three doses [37]. This was mostly because they didn't have effective ways to follow up with patients, and there were scheduling conflicts.

As part of their investigations into potential factors influencing vaccination series completion, researchers looked at the impact of school nurses on vaccine awareness and uptake. According to a recently conducted study, a significant proportion of school nurses refrained from providing parents with information about the HPV vaccine and resources, despite possessing satisfactory levels of knowledge and welcoming attitudes. This underscores the crucial necessity for school nurses to undergo specialized health promotion training, which aims to enhance their capacity to involve and enlighten parents about the advantages of HPV vaccination for their children [38].

Similarly, a small study discovered that a brief parental education program about teenage sexual health and HPV vaccination delivered by student nurses had the potential to improve sexual health outcomes. These improvements included increases in teen sexual health awareness, parental protection, and HPV vaccination initiation rates [39]. Unfortunately, the lack of research on the subject makes extrapolating and interpreting school-based barriers to HPV vaccination more difficult. Although the current state of research on this topic limits generalizability, the available findings provide useful insight into where future research efforts should focus.

Congressional work supporting HPV vaccination

On May 26, 2023, the 118th United States Congress introduced the Prevent HPV Cancers Act of 2023 (H.R3633), which amends Section 13 of the Public Health Service Act by adding a subsection titled "HPV Vaccine Public Awareness Campaign." If signed into law, the CDC would carry out this campaign with the goals of eradicating false beliefs and misinformation about HPV and the HPV vaccination, raising awareness of the importance of the HPV vaccination, and encouraging young males to complete the HPV vaccination series [3].

Studies have examined the various forms of HPV educational intervention that are currently in use and evaluated their efficacy. Sending reminder texts to parents of teenagers about their HPV vaccination status is one method that has been gaining attention [40]. Research indicates that this type of intervention not only increases the willingness of unvaccinated individuals to receive the HPV vaccine [41, 42] but also increases the likelihood of completing the series of shots [43]. Furthermore, we compared traditional generic text message reminders to precision-style messaging, which included stage-specific informational messages, clinic site walk-in hours, and the deadline for the next vaccination step. The findings revealed that both types of messages had a similar impact on HPV vaccination completion and resulted in higher vaccination rates than groups that received no messages [42]. This suggests that keeping this issue at the forefront of one's mind is essential to receiving and finishing the vaccination series.

Raising awareness and educating people about the issue leads to higher vaccination rates, reducing HPV incidence and related illnesses, some of which are life-threatening.

**Figure 2:**
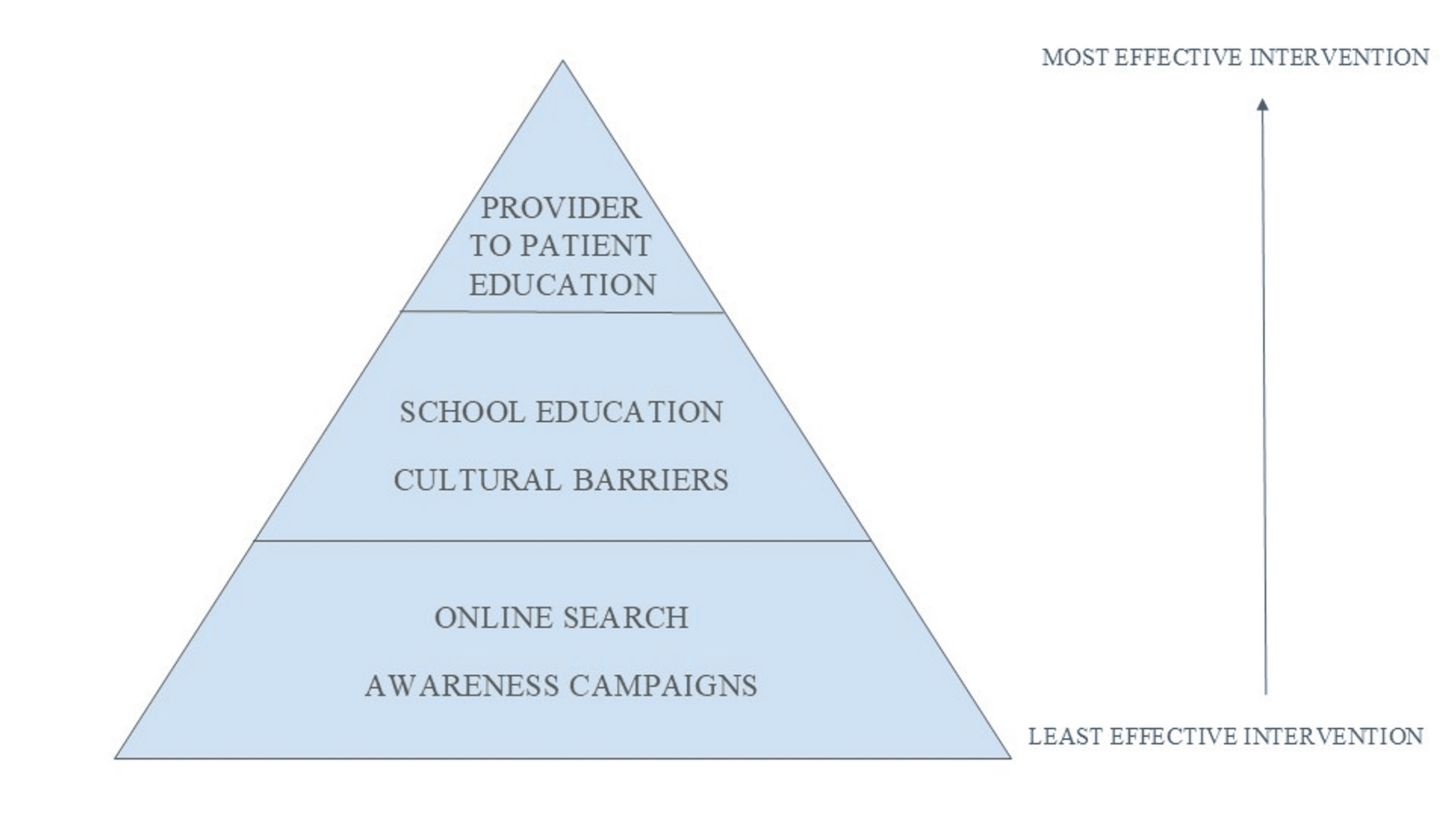
Illustration of the importance of interventions in HPV vaccine decision-making Medical provider recommendations (e.g., family physician, pediatrician) have the greatest influence on HPV vaccine decisions. Online searches and awareness campaigns are least effective [3,24].

According to the U.S. Congress's proposed campaign, resources for communities in need of HPV education should be culturally and linguistically appropriate. The materials should also highlight the safety of the vaccine, the advantages of vaccination, including the prevention of HPV-related cancers, and the target age group for the HPV vaccine. This information should be accessible to reputable health experts, healthcare providers, public health departments, and schools via a variety of channels, including social media, print, online, and in-person [3]. Healthcare professionals' actions and community involvement in HPV education significantly reduce the number of HPV cases, particularly among underrepresented groups [40]. With these policies in place, individuals from a wide range of socioeconomic and cultural backgrounds will have access to HPV education in a way that makes sense to them. To get more people vaccinated against HPV, we need to work together on politics, health care, education, and raising awareness. This will lower the number of cancers linked to HPV (Figure 2).

## Conclusions

HPV vaccination requires a diverse approach that considers politics, culture, education, and healthcare. Despite legislative initiatives and HPV vaccine licensure, the vaccination rate remains low, resulting in the transmission of this preventable disease and the spreading of related cancers. The participation of local political, religious, and community leaders is crucial for HPV vaccine education success. HPV vaccination, stigma reduction, and myth dispelling require effective communication, political leadership, and community-based activities. Using standardized HPV vaccine vocabulary, highlighting its relevance, and offering extensive information can increase comprehension and decision-making. Increased HPV vaccination rates will reduce HPV-related cancers with such a coordinated approach.
